# Aqua­(5,10,15,20-tetra­phenyl­porphyrin­ato-κ^4^ *N*)cadmium(II)–18-crown-6 (1/1)

**DOI:** 10.1107/S160053681301489X

**Published:** 2013-06-08

**Authors:** Hamza Toumi, Yassine Belghith, Jean-Claude Daran, Habib Nasri

**Affiliations:** a Laboratoire de Physico-chimie des Matériaux, Faculté des Sciences de Monastir, Avenue de l’environnement, 5019 Monastir, University of Monastir, Tunisia; b Laboratoire de Chimie de Coordination CNRS UPR 8241, 205 Route de Norbone, 31077, Toulouse, Cedex 04, France

## Abstract

The title compound, [Cd(C_44_H_28_N_4_)(H_2_O)]·(C_12_H_24_O_6_), was made by the reaction of the [Cd(TPP)] with an excess of 18-crown-6 in chloro­benzene (where TPP is tetra­phenyl­porphyrinate). The Cd^II^ cation is chelated by a TPP anion and coordinated by a water mol­ecule in a distorted N_4_O square-pyramidal geometry, the Cd^II^ cation being displaced by 0.7533 (9) Å from the mean plane of four N atoms of TPP anion. The porphyrin core presents a significant distortion, the maximum atomic deviation from the 24-atom mean plane is 0.1517 (2) Å. The 18-crown-6 mol­ecule is linked with the Cd^II^ complex *via* classical O—H⋯O hydrogen bonds. In the crystal, weak C—H⋯π inter­actions link the complex and 18-crown-6 mol­ecules into a three-dimensional supra­molecular architecture.

## Related literature

For the synthesis, see: Rodesiler *et al.* (1985*b* ▶). For related structures, see: Byrn *et al.* (1991 ▶); Ezzayani *et al.* (2013 ▶); Rodesiler *et al.* (1985*a* ▶); Mansour *et al.* (2010 ▶); Yang *et al.* (2003 ▶); Maldonado *et al.* (2009 ▶). For bond lengths in Cd^II^ complexes, see: Allen (2002 ▶). For further details of geometric distortions in related compounds, see: Scheidt & Lee (1987 ▶); Jentzen *et al.* (1997 ▶).
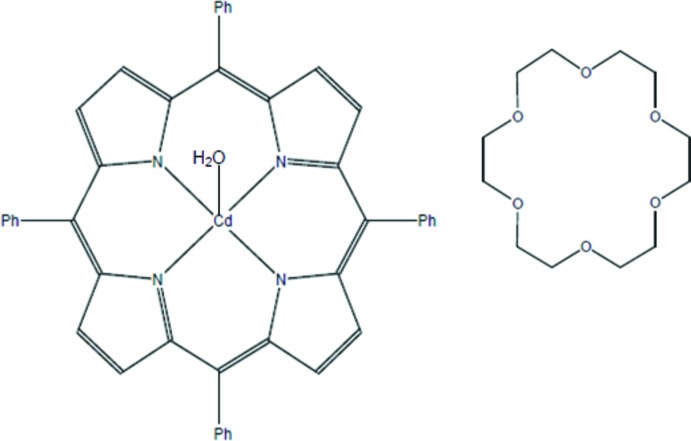


## Experimental

### 

#### Crystal data

[Cd(C_44_H_28_N_4_)(H_2_O)]·C_12_H_24_O_6_
*M* *_r_* = 1007.42
Monoclinic, 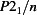

*a* = 17.1956 (2) Å
*b* = 17.0918 (2) Å
*c* = 17.3903 (2) Å
β = 106.416 (1)°
*V* = 4902.72 (10) Å^3^
*Z* = 4
Mo *K*α radiation
μ = 0.50 mm^−1^
*T* = 173 K
0.48 × 0.40 × 0.30 mm

#### Data collection

Agilent Xcalibur (Eos, Gemini ultra) diffractometer
Absorption correction: multi-scan (*CrysAlis PRO*; Agilent, 2012 ▶) *T*_min_ = 0.959, *T*_max_ = 1.000
53048 measured reflections
10009 independent reflections
8403 reflections with *I* > 2σ(*I*)
*R*_int_ = 0.027

#### Refinement

*R*[*F* ^2^ > 2σ(*F* ^2^)] = 0.028
*wR*(*F* ^2^) = 0.074
*S* = 1.04
10009 reflections
619 parameters
2 restraints
H atoms treated by a mixture of independent and constrained refinement
Δρ_max_ = 0.64 e Å^−3^
Δρ_min_ = −0.53 e Å^−3^

### 

Data collection: *CrysAlis PRO* (Agilent, 2012 ▶); cell refinement: *CrysAlis PRO*; data reduction: *CrysAlis PRO*; program(s) used to solve structure: *SIR2004* (Burla *et al.*, 2005 ▶); program(s) used to refine structure: *SHELXL97* (Sheldrick, 2008 ▶); molecular graphics: *ORTEPIII* (Burnett & Johnson, 1996 ▶) and *ORTEP-3 for Windows* (Farrugia, 2012 ▶); software used to prepare material for publication: *WinGX* (Farrugia, 2012 ▶).

## Supplementary Material

Crystal structure: contains datablock(s) I, global. DOI: 10.1107/S160053681301489X/xu5709sup1.cif

Structure factors: contains datablock(s) I. DOI: 10.1107/S160053681301489X/xu5709Isup2.hkl

Additional supplementary materials:  crystallographic information; 3D view; checkCIF report

## Figures and Tables

**Table 1:** Selected bond lengths (Å)

Cd—N1	2.2296 (15)
Cd—N2	2.2296 (15)
Cd—N3	2.2322 (16)
Cd—N4	2.2265 (15)
Cd—O1	2.2368 (18)

**Table 2:** Hydrogen-bond geometry (Å, °) *Cg*2, *Cg*3, *Cg*4 and *Cg*11 are the centroids of the N2/C6–C9, N3/C11–C14, N4/C16–C19 and C33–C38 rings, respectively.

*D*—H⋯*A*	*D*—H	H⋯*A*	*D*⋯*A*	*D*—H⋯*A*
O1—H1*O*1⋯O4	1.01 (2)	2.06 (2)	3.057 (2)	176
O1—H2*O*1⋯O6	1.00 (2)	2.04 (2)	3.013 (2)	165
C31—H31⋯*Cg*3^i^	0.95	2.93	3.651 (2)	133
C41—H41⋯*Cg*11^ii^	0.95	2.91	3.794 (2)	154
C44—H44⋯*Cg*2^iii^	0.95	2.95	3.648 (2)	131
C47—H47*A*⋯*Cg*2	0.99	2.91	3.898 (3)	173
C54—H54*B*⋯*Cg*4	0.99	2.98	3.971 (3)	176

Symmetry codes: (i) 
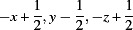
; (ii) 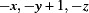; (iii) 
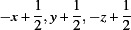
.

**Fig. 1.**
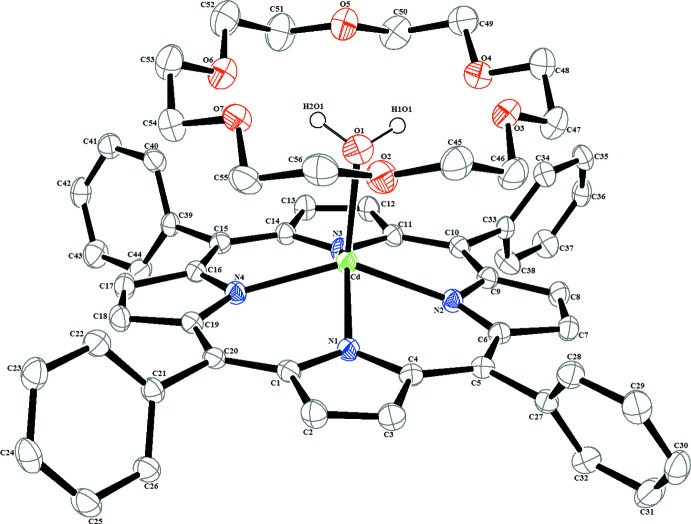
An ORTEP view of the molecular structure of the title molecule with the atom-numbering. Displacement ellipsoids are drawn at 45%. Except the two H atoms of the water axial ligand, the other H atoms have been omitted for clarity.

**Fig. 2.**
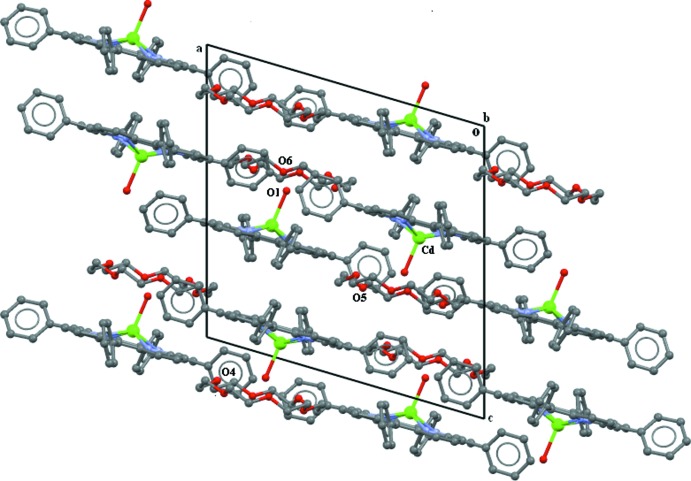
Drawing showing the packing in lattice of [CdII(TPP)(H2O)].(18-C-6), viewed down the b axis.

## supplementary crystallographic information

### Comment

In continuation of our research on the crystal structures of porphyrin complexes in general and the structures of metalloporphyrins resulting from the reaction of these species with the ether crown 18-crown-6 (Mansour *et al.*, 2010; Ezzayani *et al.*, 2013) we herein report the synthesis and crystal structure of the aqua-cadmium tetraphenylporhyrin derivative [Cd^II^(TPP)(H_2_O)].(18-C-6). The coordination geometry of the Cd^2+^ ion is square pyramidal with four Cd—*N*(pyrrole) bonds in the equatorial porphyrin plane and the Cd—O bond with a water axial ligand molecule.

The axial Cd—O(H_2_O) bond length [2.237 (2) Å] is within the range [2.210 (2) - 2.326 (1) Å] found for several cadmium-aqua non-porphyrin complexes (CSD refcodes BUYWIB10; Rodesiler *et al.*, 1985*a* and BOQQEE; Maldonado *et al.*, 2009) (CDS, version 5.34, Allen, 2002).

The average equatorial cadmium-pyrrole N atoms distance (Cd—N_p_) [2.230 (2) Å] is in the range [2.126 (9) - 2.3167 (3) Å] for Cd(II) porphyrin complexes (CSD refcodes JIVROV; Byrn *et al.*, 1991 and EXACOV; Yang *et al.*, 2003).

The cadmium atom is displaced by 0.8025 (4) Å from the 24 atoms mean plane. The porphyrin core presents a major doming deformation as seen by the positions of the N atoms above the CdN_4_C_20_ mean plane (Fig.1) ((Scheidt & Lee, 1987). This is confirmed by the Normal Structural Decomposition (NSD) calculations (Jentzen *et al.*, 1997) with a doming percentage of 47%. These calculations indicated also a saddling and ruffling distortions of the porphyrin core (~ 27% and ~ 14% respectively).

The crystal packing in the *a* and *b* directions assemble to a linear chains linked together by weak C—H···π interactions incorporating pyrrole or phenyl rings (Table 1). The parallel chains are sustained together by weak intermolecular hydrogen bonds between the O1 oxygen of the water axial ligand and the oxygene atoms of the 18-crown-6 ether crown molecule (Fig.2).

### Experimental

To a solution of [Cd(TPP)] (Rodesiler *et al.* 1985*b*) (20 mg, 0.027 mmol) in chlorobenzene (15 ml) was added an excess of 18-crown-6 (80 mg, 0.300 mmol). The reaction mixture was stirred at room temperature and at the end of the reaction, the color of the solution gradually changes from dark green to blue – purple. The resulting material was crystallized by diffusion of hexanes through the chlorobenzene solution which yields [Cd(TPP)(H_2_O)].(18-C-6). The water molecule coordinated to the cadmium come from the hygroscopic 18-crown-6 reagent used in excess.

### Refinement

All H atoms attached to C atoms were fixed geometrically and treated as riding with C—H = 0.99 Å (methylene) and 0.95 Å (aromatic) with *U*_iso_(H) = 1.2*U*_eq_(C_aromatic, methylene_). The two H atoms of the water axial ligand were found in the difference Fourier map and were included in the refinement with *U*_iso_(H) = 1.2*U*_eq_(O).

### Crystal data

**Table:**

[Cd(C_44_H_28_N_4_)(H_2_O)]·C_12_H_24_O_6_	*F*(000) = 2088
*M**_r_* = 1007.42	*D*_x_ = 1.365 Mg m^−^^3^
Monoclinic, *P*2_1_/*n*	Mo *K*α radiation, λ = 0.71073 Å
Hall symbol: -P 2yn	Cell parameters from 20195 reflections
*a* = 17.1956 (2) Å	θ = 3.0–30.2°
*b* = 17.0918 (2) Å	µ = 0.50 mm^−^^1^
*c* = 17.3903 (2) Å	*T* = 173 K
β = 106.416 (1)°	Prism, dark purple
*V* = 4902.72 (10) Å^3^	0.48 × 0.40 × 0.30 mm
*Z* = 4	

### Data collection

**Table:**

Agilent Xcalibur (Eos, Gemini ultra) diffractometer	10009 independent reflections
Radiation source: Enhance (Mo) X-ray Source	8403 reflections with *I* > 2σ(*I*)
Graphite monochromator	*R*_int_ = 0.027
Detector resolution: 16.1978 pixels mm^-1^	θ_max_ = 26.4°, θ_min_ = 3.2°
ω scans	*h* = −21→21
Absorption correction: multi-scan (*CrysAlis PRO*; Agilent, 2012)	*k* = −21→21
*T*_min_ = 0.959, *T*_max_ = 1.000	*l* = −20→21
53048 measured reflections	

### Refinement

**Table:**

Refinement on *F*^2^	Primary atom site location: structure-invariant direct methods
Least-squares matrix: full	Secondary atom site location: difference Fourier map
*R*[*F*^2^ > 2σ(*F*^2^)] = 0.028	Hydrogen site location: inferred from neighbouring sites
*wR*(*F*^2^) = 0.074	H atoms treated by a mixture of independent and constrained refinement
*S* = 1.04	*w* = 1/[σ^2^(*F*_o_^2^) + (0.0294*P*)^2^ + 3.9972*P*] where *P* = (*F*_o_^2^ + 2*F*_c_^2^)/3
10009 reflections	(Δ/σ)_max_ = 0.001
619 parameters	Δρ_max_ = 0.64 e Å^−^^3^
2 restraints	Δρ_min_ = −0.53 e Å^−^^3^

### Special details

**Table:**

Geometry. All s.u.'s (except the s.u. in the dihedral angle between two l.s. planes) are estimated using the full covariance matrix. The cell s.u.'s are taken into account individually in the estimation of s.u.'s in distances, angles and torsion angles; correlations between s.u.'s in cell parameters are only used when they are defined by crystal symmetry. An approximate (isotropic) treatment of cell s.u.'s is used for estimating s.u.'s involving l.s. planes.
Refinement. Refinement of *F*^2^ against ALL reflections. The weighted *R*-factor *wR* and goodness of fit *S* are based on *F*^2^, conventional *R*-factors *R* are based on *F*, with *F* set to zero for negative *F*^2^. The threshold expression of *F*^2^ > σ(*F*^2^) is used only for calculating *R*-factors(gt) *etc*. and is not relevant to the choice of reflections for refinement. *R*-factors based on *F*^2^ are statistically about twice as large as those based on *F*, and *R*- factors based on ALL data will be even larger.

### Fractional atomic coordinates and isotropic or equivalent isotropic displacement parameters (Å^2^)

**Table:**

	*x*	*y*	*z*	*U*_iso_*/*U*_eq_	
Cd	0.265537 (8)	0.245868 (8)	0.053198 (8)	0.02100 (5)	
N1	0.37977 (9)	0.17811 (9)	0.10136 (9)	0.0212 (3)	
N2	0.21008 (9)	0.14476 (9)	0.09930 (9)	0.0214 (3)	
N3	0.17605 (9)	0.31526 (9)	0.09616 (10)	0.0231 (3)	
N4	0.34586 (9)	0.34841 (9)	0.09638 (9)	0.0220 (3)	
O1	0.21234 (11)	0.24774 (10)	−0.07997 (11)	0.0422 (4)	
O2	0.33082 (9)	0.14168 (9)	−0.11786 (9)	0.0364 (4)	
O3	0.16534 (9)	0.08322 (9)	−0.15578 (9)	0.0352 (3)	
O4	0.03786 (9)	0.18817 (9)	−0.13841 (9)	0.0361 (4)	
O5	0.06271 (10)	0.34754 (9)	−0.17462 (9)	0.0371 (4)	
O6	0.22338 (10)	0.41108 (10)	−0.14125 (10)	0.0444 (4)	
O7	0.34991 (9)	0.30153 (9)	−0.15312 (8)	0.0322 (3)	
C1	0.45563 (11)	0.20529 (11)	0.10492 (11)	0.0211 (4)	
C2	0.50969 (12)	0.13908 (11)	0.11073 (12)	0.0271 (4)	
H2	0.5655	0.1412	0.1131	0.033*	
C3	0.46583 (12)	0.07371 (12)	0.11217 (12)	0.0271 (4)	
H3	0.4853	0.0214	0.1164	0.033*	
C4	0.38352 (11)	0.09828 (11)	0.10614 (11)	0.0216 (4)	
C5	0.31917 (11)	0.04744 (11)	0.10855 (11)	0.0218 (4)	
C6	0.23883 (11)	0.06961 (11)	0.10652 (11)	0.0213 (4)	
C7	0.17416 (12)	0.01738 (11)	0.11131 (12)	0.0248 (4)	
H7	0.1775	−0.0379	0.1164	0.030*	
C8	0.10785 (12)	0.06195 (11)	0.10721 (12)	0.0248 (4)	
H8	0.0562	0.0437	0.1089	0.030*	
C9	0.13056 (11)	0.14254 (11)	0.09982 (11)	0.0216 (4)	
C10	0.07981 (11)	0.20806 (11)	0.09666 (11)	0.0222 (4)	
C11	0.10115 (11)	0.28804 (11)	0.09593 (11)	0.0225 (4)	
C12	0.04866 (12)	0.35440 (12)	0.09641 (12)	0.0269 (4)	
H12	−0.0066	0.3522	0.0961	0.032*	
C13	0.09299 (12)	0.41978 (12)	0.09745 (12)	0.0267 (4)	
H13	0.0748	0.4722	0.0982	0.032*	
C14	0.17375 (11)	0.39514 (11)	0.09725 (11)	0.0225 (4)	
C15	0.23970 (11)	0.44530 (11)	0.10023 (11)	0.0225 (4)	
C16	0.31955 (11)	0.42321 (11)	0.10184 (11)	0.0225 (4)	
C17	0.38757 (12)	0.47598 (12)	0.11154 (12)	0.0277 (4)	
H17	0.3862	0.5313	0.1162	0.033*	
C18	0.45337 (12)	0.43202 (11)	0.11275 (12)	0.0266 (4)	
H18	0.5068	0.4507	0.1187	0.032*	
C19	0.42720 (11)	0.35104 (11)	0.10322 (11)	0.0223 (4)	
C20	0.47778 (11)	0.28538 (11)	0.10535 (11)	0.0209 (4)	
C21	0.56510 (11)	0.30243 (11)	0.11270 (11)	0.0222 (4)	
C22	0.58749 (12)	0.34409 (12)	0.05328 (12)	0.0279 (4)	
H22	0.5468	0.3618	0.0074	0.034*	
C23	0.66821 (13)	0.35997 (12)	0.06010 (13)	0.0317 (5)	
H23	0.6825	0.3885	0.0192	0.038*	
C24	0.72800 (12)	0.33410 (12)	0.12661 (14)	0.0324 (5)	
H24	0.7834	0.3442	0.1311	0.039*	
C25	0.70675 (12)	0.29358 (13)	0.18647 (13)	0.0313 (5)	
H25	0.7477	0.2763	0.2323	0.038*	
C26	0.62602 (12)	0.27795 (12)	0.18002 (12)	0.0258 (4)	
H26	0.6121	0.2504	0.2217	0.031*	
C27	0.33764 (11)	−0.03828 (11)	0.11456 (12)	0.0229 (4)	
C28	0.35747 (12)	−0.07837 (12)	0.05283 (12)	0.0269 (4)	
H28	0.3607	−0.0504	0.0066	0.032*	
C29	0.37260 (12)	−0.15827 (12)	0.05755 (14)	0.0322 (5)	
H29	0.3859	−0.1846	0.0148	0.039*	
C30	0.36830 (13)	−0.19942 (12)	0.12482 (15)	0.0353 (5)	
H30	0.3777	−0.2543	0.1279	0.042*	
C31	0.35036 (14)	−0.16063 (13)	0.18735 (14)	0.0376 (5)	
H31	0.3484	−0.1887	0.2339	0.045*	
C32	0.33515 (13)	−0.08089 (12)	0.18251 (13)	0.0319 (5)	
H32	0.3229	−0.0548	0.2259	0.038*	
C33	−0.00569 (11)	0.19123 (11)	0.09717 (11)	0.0220 (4)	
C34	−0.06227 (12)	0.16238 (12)	0.02939 (12)	0.0267 (4)	
H34	−0.0462	0.1514	−0.0175	0.032*	
C35	−0.14237 (12)	0.14941 (12)	0.02941 (13)	0.0298 (4)	
H35	−0.1805	0.1296	−0.0173	0.036*	
C36	−0.16622 (12)	0.16522 (11)	0.09690 (13)	0.0294 (4)	
H36	−0.2211	0.1576	0.0965	0.035*	
C37	−0.11035 (13)	0.19221 (14)	0.16515 (14)	0.0359 (5)	
H37	−0.1264	0.2016	0.2123	0.043*	
C38	−0.03095 (13)	0.20565 (14)	0.16522 (13)	0.0333 (5)	
H38	0.0069	0.2250	0.2123	0.040*	
C39	0.22450 (11)	0.53120 (11)	0.10536 (12)	0.0227 (4)	
C40	0.18189 (12)	0.57321 (12)	0.03822 (13)	0.0290 (4)	
H40	0.1609	0.5470	−0.0115	0.035*	
C41	0.16974 (13)	0.65313 (12)	0.04310 (14)	0.0327 (5)	
H41	0.1409	0.6812	−0.0034	0.039*	
C42	0.19919 (13)	0.69193 (12)	0.11491 (14)	0.0322 (5)	
H42	0.1910	0.7467	0.1180	0.039*	
C43	0.24083 (15)	0.65080 (13)	0.18261 (14)	0.0385 (5)	
H43	0.2606	0.6771	0.2324	0.046*	
C44	0.25354 (14)	0.57107 (13)	0.17745 (13)	0.0348 (5)	
H44	0.2826	0.5432	0.2240	0.042*	
C45	0.30525 (15)	0.06615 (13)	−0.14746 (15)	0.0386 (5)	
H45A	0.3508	0.0288	−0.1298	0.046*	
H45B	0.2878	0.0669	−0.2068	0.046*	
C46	0.23605 (15)	0.04059 (13)	−0.11659 (14)	0.0386 (5)	
H46A	0.2261	−0.0161	−0.1265	0.046*	
H46B	0.2499	0.0497	−0.0581	0.046*	
C47	0.09835 (15)	0.06200 (14)	−0.12725 (15)	0.0410 (6)	
H47A	0.1121	0.0705	−0.0686	0.049*	
H47B	0.0855	0.0059	−0.1381	0.049*	
C48	0.02653 (15)	0.11097 (14)	−0.16878 (15)	0.0402 (5)	
H48A	0.0200	0.1115	−0.2272	0.048*	
H48B	−0.0232	0.0884	−0.1598	0.048*	
C49	−0.02503 (14)	0.23930 (14)	−0.18003 (15)	0.0381 (5)	
H49A	−0.0770	0.2231	−0.1711	0.046*	
H49B	−0.0309	0.2369	−0.2383	0.046*	
C50	−0.00474 (15)	0.32122 (15)	−0.15018 (16)	0.0432 (6)	
H50A	−0.0518	0.3560	−0.1724	0.052*	
H50B	0.0084	0.3224	−0.0910	0.052*	
C51	0.08384 (17)	0.42528 (14)	−0.15134 (18)	0.0485 (6)	
H51A	0.1002	0.4292	−0.0921	0.058*	
H51B	0.0365	0.4599	−0.1727	0.058*	
C52	0.15181 (17)	0.45058 (15)	−0.18254 (17)	0.0482 (6)	
H52A	0.1387	0.4389	−0.2406	0.058*	
H52B	0.1599	0.5078	−0.1753	0.058*	
C53	0.29070 (16)	0.42850 (14)	−0.17172 (16)	0.0449 (6)	
H53A	0.3041	0.4849	−0.1646	0.054*	
H53B	0.2764	0.4163	−0.2297	0.054*	
C54	0.36231 (15)	0.38056 (14)	−0.12745 (15)	0.0416 (6)	
H54A	0.4121	0.4011	−0.1381	0.050*	
H54B	0.3692	0.3838	−0.0691	0.050*	
C55	0.41553 (14)	0.25217 (14)	−0.11332 (14)	0.0374 (5)	
H55A	0.4234	0.2542	−0.0547	0.045*	
H55B	0.4660	0.2704	−0.1242	0.045*	
C56	0.39737 (13)	0.16979 (14)	−0.14272 (14)	0.0381 (5)	
H56A	0.3841	0.1685	−0.2019	0.046*	
H56B	0.4454	0.1362	−0.1205	0.046*	
H1O1	0.1543 (7)	0.2301 (14)	−0.0972 (15)	0.046*	
H2O1	0.2057 (15)	0.3017 (8)	−0.1024 (14)	0.046*	

### Atomic displacement parameters (Å^2^)

**Table:**

	*U*^11^	*U*^22^	*U*^33^	*U*^12^	*U*^13^	*U*^23^
Cd	0.01691 (8)	0.02131 (8)	0.02469 (8)	−0.00051 (5)	0.00575 (5)	0.00129 (5)
N1	0.0182 (8)	0.0208 (8)	0.0237 (8)	−0.0008 (6)	0.0044 (6)	0.0015 (6)
N2	0.0179 (8)	0.0212 (8)	0.0252 (8)	−0.0014 (6)	0.0064 (6)	0.0007 (6)
N3	0.0193 (8)	0.0214 (8)	0.0298 (9)	−0.0019 (6)	0.0089 (7)	−0.0019 (7)
N4	0.0184 (8)	0.0210 (8)	0.0257 (8)	−0.0005 (6)	0.0048 (6)	0.0010 (6)
O1	0.0450 (10)	0.0423 (10)	0.0376 (9)	−0.0045 (8)	0.0090 (8)	0.0027 (7)
O2	0.0369 (9)	0.0359 (8)	0.0366 (9)	−0.0010 (7)	0.0110 (7)	−0.0037 (7)
O3	0.0394 (9)	0.0319 (8)	0.0336 (8)	−0.0028 (7)	0.0090 (7)	0.0072 (6)
O4	0.0375 (9)	0.0368 (8)	0.0325 (8)	−0.0023 (7)	0.0077 (7)	0.0000 (7)
O5	0.0410 (9)	0.0326 (8)	0.0431 (9)	0.0008 (7)	0.0208 (7)	−0.0011 (7)
O6	0.0474 (10)	0.0411 (9)	0.0446 (10)	−0.0062 (8)	0.0126 (8)	0.0076 (8)
O7	0.0298 (8)	0.0358 (8)	0.0285 (8)	−0.0037 (6)	0.0042 (6)	−0.0007 (6)
C1	0.0182 (9)	0.0256 (9)	0.0189 (9)	−0.0012 (7)	0.0040 (7)	−0.0002 (7)
C2	0.0192 (10)	0.0261 (10)	0.0352 (11)	0.0016 (8)	0.0064 (8)	−0.0005 (8)
C3	0.0211 (10)	0.0243 (10)	0.0342 (11)	0.0051 (8)	0.0050 (8)	0.0009 (8)
C4	0.0201 (9)	0.0217 (9)	0.0217 (9)	0.0019 (7)	0.0040 (7)	0.0017 (7)
C5	0.0214 (9)	0.0224 (9)	0.0203 (9)	0.0005 (7)	0.0037 (7)	0.0011 (7)
C6	0.0223 (9)	0.0223 (9)	0.0190 (9)	−0.0008 (7)	0.0056 (7)	0.0003 (7)
C7	0.0254 (10)	0.0210 (9)	0.0280 (10)	−0.0030 (8)	0.0076 (8)	0.0011 (8)
C8	0.0225 (10)	0.0254 (10)	0.0284 (10)	−0.0053 (8)	0.0103 (8)	0.0010 (8)
C9	0.0202 (9)	0.0238 (9)	0.0213 (9)	−0.0018 (7)	0.0068 (7)	0.0004 (7)
C10	0.0206 (9)	0.0258 (10)	0.0214 (9)	−0.0024 (8)	0.0075 (8)	−0.0015 (7)
C11	0.0197 (9)	0.0250 (10)	0.0233 (10)	−0.0019 (8)	0.0069 (8)	−0.0017 (8)
C12	0.0194 (10)	0.0275 (10)	0.0351 (11)	0.0005 (8)	0.0099 (8)	−0.0007 (8)
C13	0.0240 (10)	0.0229 (9)	0.0349 (11)	0.0022 (8)	0.0107 (9)	−0.0014 (8)
C14	0.0204 (9)	0.0218 (9)	0.0254 (10)	−0.0002 (7)	0.0065 (8)	−0.0006 (7)
C15	0.0217 (9)	0.0223 (9)	0.0233 (10)	0.0013 (7)	0.0058 (8)	0.0005 (7)
C16	0.0201 (9)	0.0221 (9)	0.0238 (10)	−0.0009 (7)	0.0038 (8)	0.0021 (7)
C17	0.0249 (10)	0.0213 (9)	0.0360 (11)	−0.0030 (8)	0.0073 (9)	0.0028 (8)
C18	0.0196 (10)	0.0256 (10)	0.0345 (11)	−0.0040 (8)	0.0074 (8)	0.0020 (8)
C19	0.0187 (9)	0.0247 (10)	0.0219 (9)	−0.0017 (7)	0.0032 (7)	0.0019 (7)
C20	0.0172 (9)	0.0249 (9)	0.0191 (9)	−0.0014 (7)	0.0025 (7)	0.0014 (7)
C21	0.0170 (9)	0.0231 (9)	0.0261 (10)	−0.0010 (7)	0.0051 (7)	−0.0011 (8)
C22	0.0223 (10)	0.0309 (10)	0.0293 (11)	−0.0004 (8)	0.0051 (8)	0.0035 (8)
C23	0.0278 (11)	0.0306 (11)	0.0394 (12)	−0.0032 (9)	0.0139 (9)	0.0041 (9)
C24	0.0180 (10)	0.0322 (11)	0.0472 (13)	−0.0034 (8)	0.0097 (9)	−0.0044 (10)
C25	0.0195 (10)	0.0349 (11)	0.0357 (12)	0.0010 (9)	0.0014 (9)	−0.0008 (9)
C26	0.0207 (10)	0.0297 (10)	0.0258 (10)	0.0000 (8)	0.0048 (8)	0.0009 (8)
C27	0.0166 (9)	0.0223 (9)	0.0275 (10)	−0.0002 (7)	0.0023 (8)	0.0021 (8)
C28	0.0219 (10)	0.0264 (10)	0.0315 (11)	0.0019 (8)	0.0059 (8)	0.0029 (8)
C29	0.0247 (11)	0.0272 (10)	0.0441 (13)	0.0032 (8)	0.0088 (9)	−0.0032 (9)
C30	0.0268 (11)	0.0201 (10)	0.0567 (15)	0.0021 (8)	0.0077 (10)	0.0052 (10)
C31	0.0378 (13)	0.0307 (11)	0.0427 (13)	0.0030 (10)	0.0086 (10)	0.0135 (10)
C32	0.0342 (12)	0.0309 (11)	0.0290 (11)	0.0015 (9)	0.0067 (9)	0.0041 (9)
C33	0.0202 (9)	0.0186 (9)	0.0284 (10)	−0.0013 (7)	0.0089 (8)	0.0001 (7)
C34	0.0257 (10)	0.0272 (10)	0.0291 (11)	−0.0024 (8)	0.0109 (8)	−0.0040 (8)
C35	0.0232 (10)	0.0270 (10)	0.0368 (12)	−0.0047 (8)	0.0046 (9)	−0.0053 (9)
C36	0.0217 (10)	0.0221 (10)	0.0473 (13)	−0.0016 (8)	0.0145 (9)	0.0007 (9)
C37	0.0322 (12)	0.0450 (13)	0.0370 (12)	−0.0047 (10)	0.0206 (10)	−0.0057 (10)
C38	0.0270 (11)	0.0458 (13)	0.0281 (11)	−0.0070 (10)	0.0096 (9)	−0.0088 (10)
C39	0.0184 (9)	0.0210 (9)	0.0306 (10)	−0.0020 (7)	0.0102 (8)	0.0004 (8)
C40	0.0268 (11)	0.0250 (10)	0.0317 (11)	−0.0002 (8)	0.0024 (9)	−0.0031 (8)
C41	0.0263 (11)	0.0257 (10)	0.0428 (13)	0.0032 (8)	0.0046 (9)	0.0055 (9)
C42	0.0296 (11)	0.0214 (10)	0.0504 (14)	−0.0008 (8)	0.0193 (10)	−0.0024 (9)
C43	0.0529 (15)	0.0298 (11)	0.0347 (12)	−0.0065 (10)	0.0154 (11)	−0.0078 (9)
C44	0.0456 (13)	0.0292 (11)	0.0282 (11)	−0.0024 (10)	0.0083 (10)	0.0034 (9)
C45	0.0467 (14)	0.0291 (11)	0.0405 (13)	0.0064 (10)	0.0131 (11)	−0.0001 (10)
C46	0.0492 (14)	0.0264 (11)	0.0397 (13)	0.0029 (10)	0.0116 (11)	0.0038 (9)
C47	0.0470 (14)	0.0343 (12)	0.0437 (14)	−0.0072 (11)	0.0161 (11)	0.0073 (10)
C48	0.0406 (13)	0.0373 (13)	0.0425 (13)	−0.0105 (10)	0.0113 (11)	0.0016 (10)
C49	0.0294 (12)	0.0475 (14)	0.0398 (13)	0.0004 (10)	0.0136 (10)	0.0032 (10)
C50	0.0400 (14)	0.0463 (14)	0.0510 (15)	0.0061 (11)	0.0252 (12)	0.0022 (11)
C51	0.0558 (16)	0.0314 (12)	0.0638 (17)	0.0038 (11)	0.0256 (14)	−0.0040 (12)
C52	0.0582 (17)	0.0318 (12)	0.0572 (16)	0.0004 (12)	0.0207 (13)	0.0022 (11)
C53	0.0519 (15)	0.0332 (12)	0.0506 (15)	−0.0068 (11)	0.0163 (12)	0.0047 (11)
C54	0.0416 (14)	0.0375 (13)	0.0428 (14)	−0.0119 (11)	0.0073 (11)	−0.0024 (10)
C55	0.0256 (11)	0.0472 (14)	0.0342 (12)	−0.0020 (10)	−0.0001 (9)	0.0015 (10)
C56	0.0251 (11)	0.0465 (13)	0.0406 (13)	0.0070 (10)	0.0059 (10)	0.0021 (10)

### Geometric parameters (Å, º)

**Table:**

Cd—N1	2.2296 (15)	C25—C26	1.387 (3)
Cd—N2	2.2296 (15)	C25—H25	0.9500
Cd—N3	2.2322 (16)	C26—H26	0.9500
Cd—N4	2.2265 (15)	C27—C28	1.395 (3)
Cd—O1	2.2368 (18)	C27—C32	1.399 (3)
N1—C4	1.367 (2)	C28—C29	1.388 (3)
N1—C1	1.370 (2)	C28—H28	0.9500
N2—C6	1.369 (2)	C29—C30	1.385 (3)
N2—C9	1.370 (2)	C29—H29	0.9500
N3—C14	1.366 (2)	C30—C31	1.381 (3)
N3—C11	1.368 (2)	C30—H30	0.9500
N4—C16	1.368 (2)	C31—C32	1.386 (3)
N4—C19	1.371 (2)	C31—H31	0.9500
O1—H1O1	1.004 (10)	C32—H32	0.9500
O1—H2O1	0.996 (10)	C33—C34	1.390 (3)
O2—C45	1.413 (3)	C33—C38	1.393 (3)
O2—C56	1.417 (3)	C34—C35	1.395 (3)
O3—C46	1.416 (3)	C34—H34	0.9500
O3—C47	1.424 (3)	C35—C36	1.375 (3)
O4—C48	1.414 (3)	C35—H35	0.9500
O4—C49	1.419 (3)	C36—C37	1.378 (3)
O5—C51	1.407 (3)	C36—H36	0.9500
O5—C50	1.417 (3)	C37—C38	1.384 (3)
O6—C52	1.409 (3)	C37—H37	0.9500
O6—C53	1.434 (3)	C38—H38	0.9500
O7—C54	1.420 (3)	C39—C44	1.390 (3)
O7—C55	1.422 (3)	C39—C40	1.390 (3)
C1—C20	1.420 (3)	C40—C41	1.388 (3)
C1—C2	1.450 (3)	C40—H40	0.9500
C2—C3	1.352 (3)	C41—C42	1.378 (3)
C2—H2	0.9500	C41—H41	0.9500
C3—C4	1.451 (3)	C42—C43	1.384 (3)
C3—H3	0.9500	C42—H42	0.9500
C4—C5	1.417 (3)	C43—C44	1.387 (3)
C5—C6	1.423 (3)	C43—H43	0.9500
C5—C27	1.497 (3)	C44—H44	0.9500
C6—C7	1.447 (3)	C45—C46	1.502 (3)
C7—C8	1.356 (3)	C45—H45A	0.9900
C7—H7	0.9500	C45—H45B	0.9900
C8—C9	1.447 (3)	C46—H46A	0.9900
C8—H8	0.9500	C46—H46B	0.9900
C9—C10	1.411 (3)	C47—C48	1.498 (3)
C10—C11	1.416 (3)	C47—H47A	0.9900
C10—C33	1.500 (3)	C47—H47B	0.9900
C11—C12	1.451 (3)	C48—H48A	0.9900
C12—C13	1.350 (3)	C48—H48B	0.9900
C12—H12	0.9500	C49—C50	1.500 (3)
C13—C14	1.452 (3)	C49—H49A	0.9900
C13—H13	0.9500	C49—H49B	0.9900
C14—C15	1.411 (3)	C50—H50A	0.9900
C15—C16	1.417 (3)	C50—H50B	0.9900
C15—C39	1.498 (3)	C51—C52	1.485 (4)
C16—C17	1.449 (3)	C51—H51A	0.9900
C17—C18	1.353 (3)	C51—H51B	0.9900
C17—H17	0.9500	C52—H52A	0.9900
C18—C19	1.450 (3)	C52—H52B	0.9900
C18—H18	0.9500	C53—C54	1.498 (3)
C19—C20	1.414 (3)	C53—H53A	0.9900
C20—C21	1.499 (2)	C53—H53B	0.9900
C21—C22	1.396 (3)	C54—H54A	0.9900
C21—C26	1.396 (3)	C54—H54B	0.9900
C22—C23	1.386 (3)	C55—C56	1.500 (3)
C22—H22	0.9500	C55—H55A	0.9900
C23—C24	1.385 (3)	C55—H55B	0.9900
C23—H23	0.9500	C56—H56A	0.9900
C24—C25	1.383 (3)	C56—H56B	0.9900
C24—H24	0.9500		
			
N4—Cd—N2	140.94 (6)	C31—C30—C29	119.9 (2)
N4—Cd—N1	83.42 (6)	C31—C30—H30	120.0
N2—Cd—N1	83.30 (6)	C29—C30—H30	120.0
N4—Cd—N3	83.65 (6)	C30—C31—C32	120.3 (2)
N2—Cd—N3	83.46 (6)	C30—C31—H31	119.8
N1—Cd—N3	140.15 (6)	C32—C31—H31	119.8
N4—Cd—O1	111.79 (6)	C31—C32—C27	120.9 (2)
N2—Cd—O1	106.98 (6)	C31—C32—H32	119.6
N1—Cd—O1	117.09 (6)	C27—C32—H32	119.6
N3—Cd—O1	102.71 (6)	C34—C33—C38	118.14 (18)
C4—N1—C1	107.92 (15)	C34—C33—C10	121.03 (17)
C4—N1—Cd	124.23 (12)	C38—C33—C10	120.82 (17)
C1—N1—Cd	124.58 (12)	C33—C34—C35	120.72 (19)
C6—N2—C9	107.97 (15)	C33—C34—H34	119.6
C6—N2—Cd	125.45 (12)	C35—C34—H34	119.6
C9—N2—Cd	123.56 (12)	C36—C35—C34	120.10 (19)
C14—N3—C11	108.00 (15)	C36—C35—H35	119.9
C14—N3—Cd	124.12 (12)	C34—C35—H35	119.9
C11—N3—Cd	123.76 (12)	C35—C36—C37	119.82 (19)
C16—N4—C19	108.22 (15)	C35—C36—H36	120.1
C16—N4—Cd	124.97 (12)	C37—C36—H36	120.1
C19—N4—Cd	125.15 (12)	C36—C37—C38	120.2 (2)
Cd—O1—H1O1	112.7 (15)	C36—C37—H37	119.9
Cd—O1—H2O1	112.8 (15)	C38—C37—H37	119.9
H1O1—O1—H2O1	100 (2)	C37—C38—C33	121.0 (2)
C45—O2—C56	113.19 (17)	C37—C38—H38	119.5
C46—O3—C47	111.75 (17)	C33—C38—H38	119.5
C48—O4—C49	112.29 (17)	C44—C39—C40	118.30 (18)
C51—O5—C50	112.35 (18)	C44—C39—C15	120.65 (18)
C52—O6—C53	113.09 (18)	C40—C39—C15	121.04 (17)
C54—O7—C55	112.54 (17)	C41—C40—C39	120.65 (19)
N1—C1—C20	125.29 (17)	C41—C40—H40	119.7
N1—C1—C2	108.80 (16)	C39—C40—H40	119.7
C20—C1—C2	125.88 (17)	C42—C41—C40	120.4 (2)
C3—C2—C1	107.23 (17)	C42—C41—H41	119.8
C3—C2—H2	126.4	C40—C41—H41	119.8
C1—C2—H2	126.4	C41—C42—C43	119.7 (2)
C2—C3—C4	107.29 (17)	C41—C42—H42	120.1
C2—C3—H3	126.4	C43—C42—H42	120.1
C4—C3—H3	126.4	C42—C43—C44	119.8 (2)
N1—C4—C5	126.15 (17)	C42—C43—H43	120.1
N1—C4—C3	108.74 (16)	C44—C43—H43	120.1
C5—C4—C3	125.05 (17)	C43—C44—C39	121.1 (2)
C4—C5—C6	126.64 (17)	C43—C44—H44	119.4
C4—C5—C27	116.76 (16)	C39—C44—H44	119.4
C6—C5—C27	116.60 (16)	O2—C45—C46	109.38 (18)
N2—C6—C5	125.08 (17)	O2—C45—H45A	109.8
N2—C6—C7	108.70 (16)	C46—C45—H45A	109.8
C5—C6—C7	126.21 (17)	O2—C45—H45B	109.8
C8—C7—C6	107.39 (17)	C46—C45—H45B	109.8
C8—C7—H7	126.3	H45A—C45—H45B	108.2
C6—C7—H7	126.3	O3—C46—C45	109.73 (18)
C7—C8—C9	107.17 (17)	O3—C46—H46A	109.7
C7—C8—H8	126.4	C45—C46—H46A	109.7
C9—C8—H8	126.4	O3—C46—H46B	109.7
N2—C9—C10	125.80 (17)	C45—C46—H46B	109.7
N2—C9—C8	108.76 (16)	H46A—C46—H46B	108.2
C10—C9—C8	125.40 (17)	O3—C47—C48	109.14 (18)
C9—C10—C11	127.39 (17)	O3—C47—H47A	109.9
C9—C10—C33	116.34 (16)	C48—C47—H47A	109.9
C11—C10—C33	116.22 (16)	O3—C47—H47B	109.9
N3—C11—C10	125.04 (17)	C48—C47—H47B	109.9
N3—C11—C12	108.70 (16)	H47A—C47—H47B	108.3
C10—C11—C12	126.24 (17)	O4—C48—C47	109.48 (19)
C13—C12—C11	107.30 (17)	O4—C48—H48A	109.8
C13—C12—H12	126.4	C47—C48—H48A	109.8
C11—C12—H12	126.4	O4—C48—H48B	109.8
C12—C13—C14	107.26 (17)	C47—C48—H48B	109.8
C12—C13—H13	126.4	H48A—C48—H48B	108.2
C14—C13—H13	126.4	O4—C49—C50	109.2 (2)
N3—C14—C15	125.57 (17)	O4—C49—H49A	109.8
N3—C14—C13	108.74 (16)	C50—C49—H49A	109.8
C15—C14—C13	125.67 (17)	O4—C49—H49B	109.8
C14—C15—C16	127.11 (17)	C50—C49—H49B	109.8
C14—C15—C39	116.46 (16)	H49A—C49—H49B	108.3
C16—C15—C39	116.39 (16)	O5—C50—C49	108.82 (19)
N4—C16—C15	125.82 (17)	O5—C50—H50A	109.9
N4—C16—C17	108.54 (16)	C49—C50—H50A	109.9
C15—C16—C17	125.62 (18)	O5—C50—H50B	109.9
C18—C17—C16	107.46 (17)	C49—C50—H50B	109.9
C18—C17—H17	126.3	H50A—C50—H50B	108.3
C16—C17—H17	126.3	O5—C51—C52	109.6 (2)
C17—C18—C19	107.28 (17)	O5—C51—H51A	109.7
C17—C18—H18	126.4	C52—C51—H51A	109.7
C19—C18—H18	126.4	O5—C51—H51B	109.7
N4—C19—C20	125.54 (17)	C52—C51—H51B	109.7
N4—C19—C18	108.49 (16)	H51A—C51—H51B	108.2
C20—C19—C18	125.86 (17)	O6—C52—C51	109.8 (2)
C19—C20—C1	127.07 (17)	O6—C52—H52A	109.7
C19—C20—C21	116.22 (16)	C51—C52—H52A	109.7
C1—C20—C21	116.65 (16)	O6—C52—H52B	109.7
C22—C21—C26	118.36 (17)	C51—C52—H52B	109.7
C22—C21—C20	121.01 (17)	H52A—C52—H52B	108.2
C26—C21—C20	120.62 (17)	O6—C53—C54	109.2 (2)
C23—C22—C21	121.04 (19)	O6—C53—H53A	109.8
C23—C22—H22	119.5	C54—C53—H53A	109.8
C21—C22—H22	119.5	O6—C53—H53B	109.8
C24—C23—C22	119.9 (2)	C54—C53—H53B	109.8
C24—C23—H23	120.1	H53A—C53—H53B	108.3
C22—C23—H23	120.1	O7—C54—C53	109.28 (19)
C25—C24—C23	119.80 (19)	O7—C54—H54A	109.8
C25—C24—H24	120.1	C53—C54—H54A	109.8
C23—C24—H24	120.1	O7—C54—H54B	109.8
C24—C25—C26	120.5 (2)	C53—C54—H54B	109.8
C24—C25—H25	119.8	H54A—C54—H54B	108.3
C26—C25—H25	119.8	O7—C55—C56	109.31 (17)
C25—C26—C21	120.45 (19)	O7—C55—H55A	109.8
C25—C26—H26	119.8	C56—C55—H55A	109.8
C21—C26—H26	119.8	O7—C55—H55B	109.8
C28—C27—C32	117.84 (18)	C56—C55—H55B	109.8
C28—C27—C5	121.36 (17)	H55A—C55—H55B	108.3
C32—C27—C5	120.80 (18)	O2—C56—C55	108.82 (19)
C29—C28—C27	121.3 (2)	O2—C56—H56A	109.9
C29—C28—H28	119.3	C55—C56—H56A	109.9
C27—C28—H28	119.3	O2—C56—H56B	109.9
C30—C29—C28	119.7 (2)	C55—C56—H56B	109.9
C30—C29—H29	120.1	H56A—C56—H56B	108.3
C28—C29—H29	120.1		

### Hydrogen-bond geometry (Å, º)

Cg2, Cg3, Cg4 and Cg11 are the centroids of the N2/C6–C9, N3/C11–C14, N4/C16–C19 and C33–C38 rings respectively.

**Table:**

*D*—H···*A*	*D*—H	H···*A*	*D*···*A*	*D*—H···*A*
O1—H1*O*1···O4	1.01 (2)	2.06 (2)	3.057 (2)	176
O1—H2*O*1···O5	1.00 (2)	2.55 (2)	3.138 (2)	118
O1—H2*O*1···O6	1.00 (2)	2.04 (2)	3.013 (2)	165
C31—H31···*Cg*3^i^	0.95	2.93	3.651 (2)	133
C41—H41···*Cg*11^ii^	0.95	2.91	3.794 (2)	154
C44—H44···*Cg*2^iii^	0.95	2.95	3.648 (2)	131
C47—H47*A*···*Cg*2	0.99	2.91	3.898 (3)	173
C54—H54*B*···*Cg*4	0.99	2.98	3.971 (3)	176

Symmetry codes: (i) −*x*+1/2, *y*−1/2, −*z*+1/2; (ii) −*x*, −*y*+1, −*z*; (iii) −*x*+1/2, *y*+1/2, −*z*+1/2.
